# Microperimetry and Multifocal Electroretinogram in a Patient With Unilateral Retinal Pigment Epithelium Dysgenesis (URPED)

**DOI:** 10.1155/crop/7911612

**Published:** 2025-08-14

**Authors:** Beatriz de Lucena Ribeiro, Ana Lucia Passos Peixoto, Ana Paula Couto, Rafael Erthal Robbs, Wander Borges, Julieta Micherif, Giovanna Provenzano, Raul N. G. Vianna

**Affiliations:** Department of Ophthalmology, Fluminense Federal University (UFF), Niteroi, Rio de Janeiro, Brazil

**Keywords:** microperimetry, multifocal electroretinography, unilateral retinal pigment epithelium dysgenesis, URPED

## Abstract

**Introduction:** This study is aimed at describing a patient with unilateral retinal pigment epithelium dysgenesis (URPED) using multimodal retinal imaging combined with ocular microperimetry and multifocal electroretinogram (ERG) analysis.

**Case presentation:** A 56-year-old healthy male was referred for a routine ophthalmologic control. His best corrected visual acuity was 20/20 and 20/25 in the right and left eye, respectively. Fundus examination of the left eye revealed a well-circumscribed, large yellowish-white lesion on the posterior pole that extended from the peripapillary region to the inferior temporal vascular arcade, sparing the fovea. This characteristic fundus picture led us to the diagnosis of URPED. Microperimetry demonstrated a progressive decrease of sensitivity from the normal retina toward the lesion borders, reaching a value of 0 dB at its center. Multifocal ERG displayed a reduction of central amplitudes in the involved eye.

**Discussion:** Our findings indicate a varied degree of sensitivity at the site of the lesion. Despite good visual acuity, multifocal ERG revealed reduced macular function.

## 1. Introduction

First described by Cohen et al., unilateral retinal pigment epithelium (RPE) dysgenesis (URPED) is a rare retinal disorder characterized by a unique scalloped reticular border of fibrosis and RPE atrophy encircled by pools of hyperplastic RPE commonly located at the posterior pole with optic nerve involvement [1, 2]. Diagnosis is based on the observation of the retinal findings and endorsed by the retinal multimodal analysis.

This manuscript describes a healthy male with unilateral retinal findings consistent with URPED. As a rare condition, limited information regarding URPED is available in the literature, and more studies are required for a better elucidation of this retinal entity. Thus, in an attempt to increase the knowledge regarding the visual impairment observed in URPED, a multimodal retinal analysis was performed, including fundus autofluorescence (FAF), fluorescein angiography (FA), indocyanine–green angiography (ICGA), spectral domain optical coherence tomography (SD-OCT), computerized perimetry (CP), microperimetry (MP) and multifocal ERG (mfERG).

To the best of our knowledge, MP and mfERG findings have not been previously described in the context of URPED.

## 2. Case Presentation

A 56-year-old healthy male was referred for a routine ophthalmologic control. Past medical history and ocular history were unremarkable. The best corrected visual acuity was 20/20 and 20/25 in the right and left eye, respectively. Slit-lamp examination of the anterior segment and intraocular pressure were normal in both eyes.

**Figure 1:**
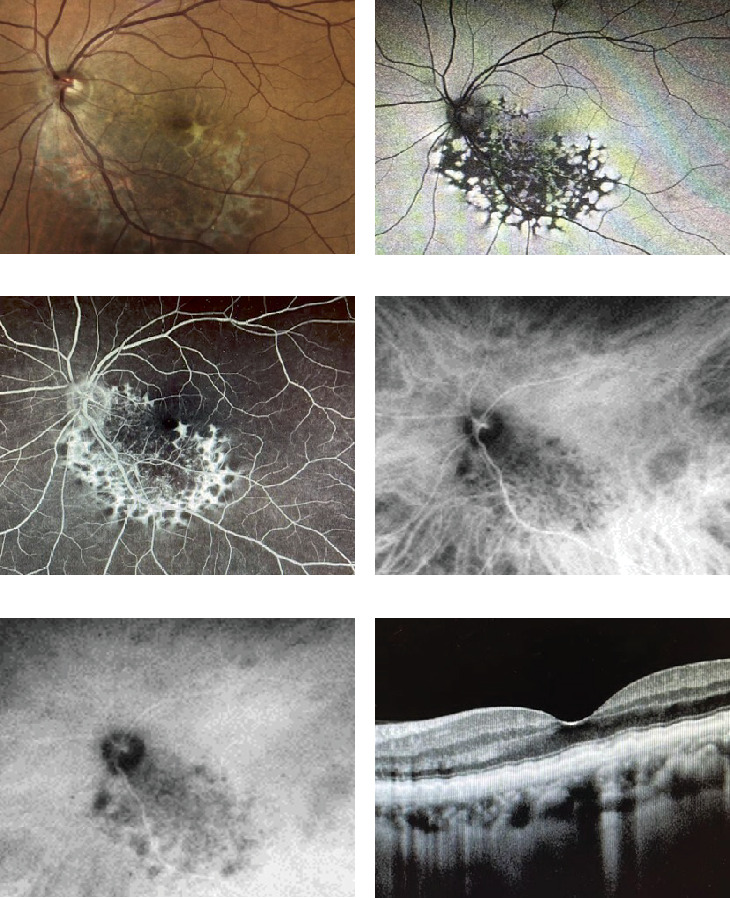
URPED in a 56-year-old man with 20/25 vision. (a) Fundus photography with a unilateral solitary lesion of the RPE with fibrosis and hyperplastic changes at the periphery of the lesion and atrophy in its center. (b) FAF indicates the RPE changes with a distinct inverted pattern with (c) FA. (d) Early phase of ICGA and (e) late phase of ICGA evidenced diffuse hypofluorescence and a discrete hyperfluorescence margin. (f) SD-OCT (horizontal scan) indicates thickening of the external retina, displaying a waveform appearance associated with atrophy of the RPE.

Fundus examination of the right eye appeared normal. However, the left eye revealed a well-circumscribed, large yellowish-white lesion on the posterior pole that extended from the peripapillary region to the inferior temporal vascular arcade, sparing the fovea (Figure 1a). Mild vascular tortuosity was observed above the lesion. FAF indicated a hypoautofluorescent lesion with a hyperautofluorescent well-carved margin, forming a reticular pattern (Figure 1b). FA revealed a “window defect” hyperfluorescent lesion outlined by a scalloped hypofluorescent margin (Figure 1c). ICGA demonstrated diffuse hypofluorescence in the early phase, that was better outlined during the late phase (Figure 1d,e). SD-OCT macular scanning of the left eye revealed a thickening of the nerve fiber layer and the external retina, displaying a waveform appearance mottled by signal hypertransmission due to atrophic RPE (Figure 1f).

**Figure 2:**
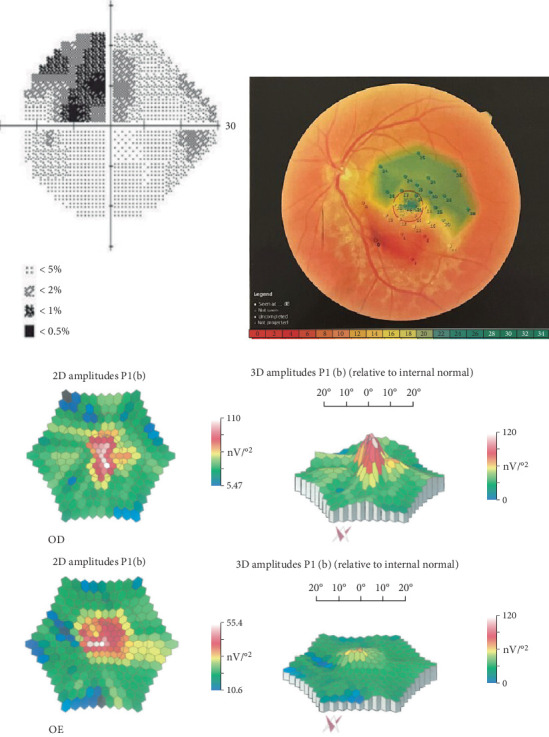
URPED in a 56-year-old man with 20/25 vision. (a) Computerized perimetry indicating increased blind spot and upper nasal scotoma. (b) Microperimetry showing a relative scotoma at the inferior region with different patterns of retinal sensitivity of 2–14 dB (yellow and light red areas) and absolute scotoma with a retinal sensitivity of 0 dB (dark red area). (c) Multifocal electroretinogram indicating a normal P1-wave amplitude in the right eye and an important reduction in the left eye.

CP (Figure 2a) indicated an increased blind spot and upper nasal scotoma in the affected eye. MP (Figure 2b) demonstrated different patterns of sensitivity from the superior normal retina (green area), with a gradual retinal sensitivity reduction of 2–14 dB (yellow and light red areas) and reaching an absolute scotoma with a retinal sensitivity of 0 dB (dark red area) in the center of the lesion. mfERG (Figure 2c) displayed an important reduction in the amplitude of the P1 wave.

The diagnosis of URPED was acknowledged, and a conservative management approach was adopted.

## 3. Discussion

URPED is a rare, idiopathic retinal disorder with variable presentation age and no systemic or other ocular associations [1]. Most patients are asymptomatic and are often diagnosed incidentally, even though visual acuity can vary due to macular involvement or other secondary complications such as choroidal neovascularization (CNV) or epiretinal membrane [3]. Fundoscopic findings typically indicate a unilateral, well-circumscribed, large yellowish-white lesion on the posterior pole with a peculiar scalloped margin of hypopigmented RPE patches outlined by hyperpigmentation. This condition presents mild progression; however, it can be associated with epiretinal fibrous proliferation, CNV, serous or tractional retinal detachment, retinal folds, and macular hole [3]. URPED is considered idiopathic but may represent a spectrum or *forme fruste* of combined hamartoma of the retina and RPE [4].

Our report details a healthy 56-year-old male with URPED. The patient presented with a lesion on the posterior pole contiguous to the optic nerve, sparing the umbus. Multimodal retinal imaging revealed the characteristic inverted pattern on FAF and FA, with central atrophic patches of RPE and a well-defined scalloped margin due to RPE hyperplasia. No associated retinal complications were observed.

SD-OCT findings revealed external retinal thickening with a waveform appearance and mottled RPE atrophy, consistent with URPED. Literature describes SD-OCT with central RPE atrophic changes, increased transmission defect, fibroglial changes, CNV, and epiretinal membrane. Attenuation of the inner segment–outer segment junction and choroidal thinning have also been reported [5].

ICGA in our case revealed diffuse hypofluorescence with a delineated hyperfluorescence margin during early and late phases, as well as focal hypofluorescent spots and these observations were consistent with the findings described by Cervera-Taulet et al. [6]

Despite preserved visual acuity, CP revealed a visual deficit confirmed by MP that indicated a sensitivity pattern characterized by a progressive moderate to severe visual sensitivity reduction toward the lesion site. mfERG indicated localized central abnormal amplitudes supporting the discrete functional deficit of the visual acuity. In the previously reported case by Yamasaki et al., the visual field test, single-flash ERG, and scotopic ERG showed normal results. The 30-Hz flicker test and photopic ERG showed a decrease in amplitude in the affected eye. However, mfERG was not performed [5].

Although the diagnoses of URPED is straightforward, some differential diagnoses can include conditions with a “leopard-spot” fundus pattern like large B-cell vitreoretinal lymphoma, uveal effusion syndrome, lymphocytic leukemia, diffuse uveal melanocytic proliferation, hypertensive choroidopathy, and acute zonal occult outer retinopathy [1, 2, 4]. A systemic evaluation with multimodal retinal analysis is essential for an accurate diagnosis.

Currently, URPED is not recognized in medical literature as being associated with specific ocular or systemic findings. However, Romano et al. have recently reported a case where URPED was associated with retinal capillary hemangioma, suggesting that this association could indicate a common developmental defect [7].

Our report provides a better understanding of the visual impairment observed in URPED. Nevertheless, more studies are necessary to expand our knowledge regarding this intriguing disorder.

## Data Availability

Data sharing is not applicable to this article as no new data were created or analyzed in this study.
